# A Fuzzy Comprehensive Assessment and Hierarchical Management System for Urban Lake Health: A Case Study on the Lakes in Wuhan City, Hubei Province, China

**DOI:** 10.3390/ijerph15122617

**Published:** 2018-11-22

**Authors:** Teng Wang, Jingjing Yan, Jinlong Ma, Fei Li, Chaoyang Liu, Ying Cai, Si Chen, Jingjing Zeng, Yu Qi

**Affiliations:** 1 Hubei Research Center of Water Affair, Hubei University of Economics, Wuhan 430073, China; wangtenghbue@163.com; 2 School of Information and Safety Engineering, Zhongnan University of Economics and Law, Wuhan 430073, China; Yanjing@zuel.edu.cn (J.Y.); majinlong@zuel.edu.cn (J.M.); lcy@zuel.edu.cn (C.L.); 1993cy@zuel.edu.cn (Y.C.); jjzeng@zuel.edu.cn (J.Z.); z0004447@zuel.edu.cn (Y.Q.); 3 School of Resources and Environment, Hubei University, Wuhan 430062, China; kathryncs123@hotmail.com

**Keywords:** urban lake, comprehensive nutrition status Index, heavy metals, health risk, fuzzy comprehensive method

## Abstract

Environmental assessment of eutrophication or heavy metals in urban lakes is an important reference for identifying the pollution degree and formulating pollution prevention strategies. At present, the most research on lake health states is often evaluated from a single angle for toxic metals pollution or eutrophication using the standard comparison method for both, the comprehensive trophic level index (TLI), and the health risk assessment for toxic metals. Moreover, the above deterministic methods probably lead to biased or unreliable assessment due to the randomness and fuzziness in environment system caused by natural change and human activities. In this paper, a fuzzy comprehensive lake health assessment method (FCLHAM) was established to evaluate comprehensive lake health states more comprehensively and accurately, which integrates quantitative eutrophication and health risk considerations. To test and verify FCLHAM, 21 lakes, scientifically selected from the total 143 lakes in the Chinese Wuhan city as study case, were investigated and analyzed for their state of eutrophication and the health risk posed by heavy metals. According to the FCLHAM, the average comprehensive lake health state decreased in the sequence of L20 (considerate risk level) > L1–L17, L19, L21 (moderate risk level) > L18 (low risk level). Based on the result, lakes were classified into three categories: general management (L18), enhanced management (L1–L17, L19, L21), and priority management (L20). If the 143 lakes in Wuhan were classified by the “area-region-function” classification, they would be assigned to the same category as the representative lakes of the same type. At this point, we will attribute all of Wuhan’s lakes to the three types. Depending on the characteristics of each type, a targeted approach to different types of management for each type of lake is a more efficient way to manage many of Wuhan’s lakes. This management mode also serves as an effective reference for the environmental management of urban lakes both at home and abroad. In other words, according to the FCLHAM, a hierarchical management system based on lake characteristics classification was obtained.

## 1. Introduction

Eutrophication is a common water pollution phenomenon caused by excessive nutrients and is accompanied by ecological problems such as algal blooms, oxygen depletion, aquatic organism death, and aquatic ecosystem deterioration [1]. With the increase of population and rapid urbanization, nutrient loading in lots of urban lakes has increased and exceeded their corresponding natural carrying capacities [2,3,4]. Especially, urban lake eutrophication poses a serious threat to regional economic development, the ecological environment, and drinking water security [5,6]. Furthermore, heavy metal pollutants are discharged into urban lakes through industrial, agricultural, and domestic waste-water discharges, precipitation, and the release of contaminated sediment [7,8,9]. To a certain extent, toxic metal accumulation in urban lakes can do serious harm to the “water—aquatic plant—aquatic animal” system which will affect human health directly or indirectly through drinking water, food chains, etc. [10].

In recent years, various works have been done to explore the heavy metal or eutrophication distributions and sources in urban lakes [11,12], as well as toxic metal eco-risk and health risk levels [13,14], environmental management strategies [15,16], and corresponding remediation technologies [17]. Obviously, eutrophication and heavy metal pollution play key roles in urban lake health. Environmental assessment of eutrophication or heavy metals in urban lakes is an important reference for identifying the degree of pollution and formulating pollution prevention strategies. Unfortunately, most research on lake risk is often evaluated from a single angle for toxic metals pollution or eutrophication using the standard comparison method [18,19,20] for both, the comprehensive trophic level index (TLI) [21,22], and the health risk assessment for toxic metals [23,24]. Moreover, the above deterministic methods probably lead to biased or unreliable assessments due to the randomness and fuzziness in environment systems caused by natural change and human activities [25,26,27,28]. Frankly, when there are different theoretical foundations, the evaluation results and conclusions differ to some extent, which makes it challenging for decision-makers to make scientific and synthetic management decision under consideration of separate assessment methods. Therefore, in order to implement appropriate environmental management strategies and measures, it is important to explore a feasible lake health assessment method synthetically considering lake eutrophication and corresponding health risks posed by heavy metals, together with evaluation of systematic uncertainty.

The objectives of this study were (i) to investigate and analyze the state of eutrophication and the health risk posed by heavy metals in the selected lakes from the total 143 lakes in the Chinese Wuhan city as a study case; (ii) to develop a fuzzy comprehensive lake health assessment method (FCLHAM) integrating quantitative eutrophication and health risk consideration; (iii) to test and verify FCLHAM by assessing the integrated lake health states of the studied lakes; and (iv) to introduce a novel hierarchical management system for lakes based on the FCLHAM results.

## 2. Materials and Methods

### 2.1. Study Area

Known for having over 100 lakes, Wuhan city (113°41′ E–115°05′ E; 29°58′ N–31°22′ N) is located in the interior of China, in the eastern part of Jianghan Plain. Located on the northern side of the Northern Tropic, the sub-tropical monsoon humid climate zone has a mean annual temperature of 15.8 °C–17.5 °C and an annual rainfall of 1150–1450 mm. Wuhan city has 143 lakes and the total area of lakes is 803.2 km^2^, which is the highest of all Chinese cities. Because of the high ecological value and the large number of lakes, the local economy is increasing rapidly. Additionally, various functions of these lakes play significant roles in the development of the city. But, as a result of the excessive increase of economy and other factors, the number of lakes is rapidly decreasing and some lakes are polluted to a certain degree, bringing adverse effects to sustainable urban development.

### 2.2. Sample Collection and Analysis Methods

**Figure 1:**
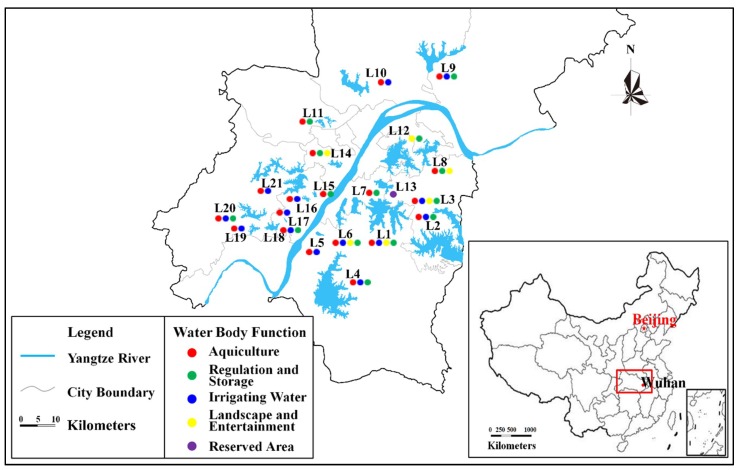
The geolocation of the 21 lakes in Wuhan (ArcGIS software version 9.3 (https://www.arcgis.com/index.html)).

One hundred and forty-three lakes in Wuhan (Figure S1) were divided into large lakes (≥20 km^2^), medium lakes (10 km^2^–20 km^2^), and small lakes (≤10 km^2^) with the proportion of 3%, 6%, and 91%, respectively. The large and medium-sized lakes were small in number and important to the development of Wuhan city, so they were all included in the typical lakes. Other typical lakes in the small lake category were selected by combing the region and function further. Based on regionalization, the lakes could be divided into Central District, Dongxihu District, Caidian District, Hannan District, Jiangxia District, Huangpi District, Xinzhou District, Economic Development Zone, and the High and New Technology Development Zone of Donghu lake. The lakes have five main water functions: regulation, irrigation, water supply, aquaculture or planting, landscape or entertainment, and reservation. Most lakes had multiple functions, especially large lakes, while small lakes were relatively simple in function. According to the preliminary classification of region and function, the small lakes were selected comprehensively to contain all city regions and functions. The selection method is presented in Figure S2. Therefore, under the premise of ensuring the results are representative and scientific, a total of 21 typical lakes (Figure 1) were screened and selected from 143 lakes in Wuhan city based on comprehensive consideration of their characteristics of area, function, and region. Figure 1 was made based on our investigation’s data and the ArcGIS software version 9.3 (Environmental Systems Research Institute, Redlands, CA, USA) (https://www.arcgis.com/index.html). The 21 selected typical lakes were Tangxun Lake (L1), Niushan Lake (L2), Baoxie Lake(L3), Luhu Lake (L4), Shenshan Lake (L5), Qingling Lake (L6), Huangjia Lake (L7), Yanxi Lake (L8), Wuhu Lake (L9), Houhu Lake (L10), Jingyin Lake (L11), Donghu Lake (L12), Nanhu Lake (L13), Moshui Lake (L14), Tanghu Lake (L15), Lanni Lake (L16), Zhongshan Lake (L17), Guanlian Lake (L18), Tonghu Lake (L19), Xiaozha Lake (L20), and Hougong Lake (L21).

The design layout and sampling methods of lake water samples were strictly according to the Chinese Technical Specifications Requirements for Monitoring of Surface Water and Waste Water (HJ91-2002) and the Chinese Water Quality Technical Regulation on the Design of Sampling Programs (HJ495-2009), combined with the area size and hydrological characteristics of the studied lake. All water samples were collected into polytetrafluoroethylene bottles which were rinsed with lake water at least three times before using. Afterwards, water samples were acidified to pH 1–2 with H_2_SO_4_ and then kept in thermostats with ice bags, and transferred to the laboratory within 24 h. The water temperature (T), pH, dissolved oxygen (DO), electrical conductivity (EC), and Chl-*a* were measured by a portable water quality analyzer (HQ40d, HACH, Loveland, CO, USA) and the transparency (SD) was measured by the lead method in situ. In the laboratory, the concentration of TP was measured by Molybdenum Antimony Spectrophotometry (GB11893-89, China), the concentration of TN was measured by Alkaline Potassium Persulfate Digestion UV Spectrophotometry (HJ636-2012, China), and the concentration of COD_Mn_ was measured by Water Quality—Determination of Permanganate Index (GB11892-89, China). In addition, the total amounts of Cu, Zn, Cr, Cd, Mn, Fe, Ni, and Pb were measured with Atomic Absorption Spectroscopy (AAS ZEEnit 700P, Jena, Germany), the total amount of As and Hg were measured by Atomic Fluorescence Spectrometry (AFS-9730, Haiguang Instrument Co. Ltd., Beijing, China), both of the determinations were according to Water Quality—Digestion of Total Metals-Nitric Acid Digestion Method (HJ677–2013, China) and Water Quality—Determination of Mercury, Arsenic, Selenium, Bismuth and Antimony—Atomic Fluorescence Spectrometry (HJ694-2014, China).

To ensure the accuracy and reliability of analysis, parallel samples and blank samples were used to analyze error and at least 10% of each batch of samples was taken as parallel samples. If the relative deviation of the results was less than 20%, the analysis results were considered reliable. The analysis results of blank samples should be lower than the detection limits, so as to eliminate the pollution that might generate between processing procedures and determinations. The standard curve would be drawn for each sample analysis, and the correlation coefficient of the standard curve was not below 0.995.

### 2.3. Comprehensive Trophic Level Index (TLI) Method

TLI is one of the comprehensive eutrophication evaluation methods taking chlorophyll a (Chl-*a*), total phosphorus (TP), total nitrogen (TN), transparency (SD), and permanganate index (COD_Mn_) as the evaluation indicators [28,29]. It was widely used in trophic state assessment of lakes and rivers due to its diversity and applicability in evaluation indicators [30,31]. TLI takes Chl-*a* as the benchmark parameter, obtaining the corresponding weights of all parameters depending on the correlation degree between the benchmark parameter and the other parameters, and then obtains the TLI by a weighted algorithm [29]. The TLI model is as follows [32]:

(1) $$TLI\left(\Sigma \right)=\sum_{j=1}^{n}W_{j}\times TLI\left(j\right)\;$$

where *TLI*(Ʃ) is comprehensive trophic level index. *W_j_* represents the corresponding weight of parameter *j. TLI*(Ʃ) represents trophic state index of parameter *j. n* is the numbers of evaluation parameters.

With Chl-*a* as the benchmark parameter, the normalized correlation weight of parameter *j* is as follows [32]:

(2) $$W_{j}=r_{ij}{}^{2}/\sum_{j=1}^{n}r_{ij}{}^{2}\;$$

where *W_j_* represents the corresponding weight of parameter *j. r_ij_* represents the correlation coefficient between benchmark parameter and parameter *j. n* is the numbers of evaluation parameters. Based on a eutrophication survey of Chinese lakes, the correlation coefficients *r_ij_* between the benchmark parameter (Chl-*a*) and other parameters are *r*_Chl-*a*_ = 1, *r*_TN_ = 0.82, *r*_TP_ = 0.84, *r*_SD_ = 0.83 and *r_CODMn_* = 0.83 [28,32].

Trophic level indexes of each parameter are calculated as Equations (3)–(7).

(3) $$TLI(\mathrm{Chl}-\alpha )=10\times (2.5+1.086\ln\rho_{\mathrm{Chl}-\alpha })\;$$

(4) $$TLI\left(TP\right)=10\times \left(9.436+1.624\ln\rho_{TP}\right)\;$$

(5) $$TLI\left(TN\right)=\left(10\times 5.453+1.694\ln\rho_{TN}\right)\;$$

(6) $$TLI\left(SD\right)=10\times \left(5.118-1.94\ln\rho_{SD}\right)\;$$

(7) $$TLI\left(COD{}_{MN}\right)=10\times \left(0.109+2.66\ln\rho_{COD{}_{MN}}\right)\;$$

where *ρ*_Chl*-a*_ represents concentration of Chl*-a* (mg/m^3^), and *ρ_TP_ ρ_TN_ ρ*_*COD_Mn_*_ represent concentrations of *TP*, *TN*, and *COD_Mn_* (mg/L), respectively. *ρ_SD_* represents transparency (m). The trophic state of the lakes is graded using continuous numbers from 0 to 100, as shown in Table 1 [28,32].

### 2.4. Health Risk for Heavy Metals in Lakes

Health risk assessment is described as processes used to estimate event probability and probable degree of adverse health effects over a specific period [33,34,35]. Risk level of environmental pollutants to human beings depends on the body’s exposure dose to the pollutants and the toxicity of the pollutants. There are two main pathways for human exposure to trace elements in water: ingestion and dermal absorption, ignoring exposure via inhalation [35,36]. The exposure dose can be calculated by Equations (8) and (9) [37,38].

(8) $$ADD_{ing}=\frac{C_{W}\times IR\times EF\times ED}{BW\times AT}\;$$

where *ADD_ing_* (µg/(kg·day)) represents the exposure dose through ingestion. In this study, the ingestion mainly refers to the intake through water from studied lakes. *C_w_* is the mean concentration of trace element in water (µg/L). *IR* is the intake rate of water, including direct drinking rate and indirect drinking rate (L/day). *EF* is the exposure frequency to pollutants (day/year). *ED* is the exposure duration, and it means the length of time over which contact with the contaminant lasts (year). *BW* represents the body weight (kg). *AT* is the average time (day). For carcinogenic risk, *AT* is the average life expectancy of people [37,38].

(9) $$ADD_{derm}=\frac{C_{W}\times SA\times K_{P}\times ET\times EF\times ED\times 10^{-3}}{BW\times AT}\;$$

where *ADD_derm_* (µg/(kg·day)) represents the exposure dose through dermal absorption. *SA* is the exposure area of skin (cm^2^). *K_p_* is the dermal permeability coefficient of pollutants in water (cm/h), in this study, 0.001 cm/h for Cu, Cd, and As, 0.0001 cm/h for Pb, 0.002 cm/h for Cr, and 0.0006 cm/h for Zn [14,35], and *ET* is the exposure time (h/day). In this study, *ET* is 0.6 h/day. For the meanings of *C_w_*, *EF*, *ED*, *BW*, and *AT*, please refer to Equation (8).

The health risks caused by environmental pollutants can be divided into carcinogenic risk and non-carcinogenic risks according to their properties. In general, carcinogens are of greater risk than non-carcinogens. Therefore, the risk of cancer caused by lake water is used as the assessment medium of lake health risk.

Carcinogenic risk is the product of daily exposure dose and cancer slope factor, which is shown in Equation (10). Under the assumption that there is no antagonism and synergism between pollutants, the integrated carcinogenic risk can also be identified as the sum of carcinogenic risks exposure by various pollutants via different pathways as shown in Equation (11). The EPA believes that carcinogenic risk value of human being is acceptable within 1 × 10^−4^, while the maximum acceptable risk value recommended by International Commission on Radiological Protection (ICRP) is 5 × 10^−5^ [14]. The significant difference between the two evaluation standards may mislead the decision makers in their final judgment. Furthermore, it should be noted that there is currently no official and uniform standard of acceptable risk value in China and many developing countries, which may lead to uncertainty and incomparability among different decision-makers. Therefore, risk classification was carried out in this study in order to make the evaluation results clearer and more intelligible. Risk levels were rated as five levels based on the Delphi method, assessment criteria of USEPA and ICRP, as well as existing research (Table 2) [38].

(10) $$CR_{i}=ADD_{i}\times CSF_{i}\;$$

(11) $$CR=\sum_{i}^{n}CR_{i}\;$$

where *CR_i_* is the carcinogenic risk of trace elements through ingestion or dermal absorption, dimensionless. *ADD_i_* (µg/(kg·day)) is the daily exposure dose of carcinogenic pollutants. *CSF_i_* (kg·day/µg) is the cancer slope factor of carcinogenic pollutants. *CR* is the sum of *CR_i_. i* is the pathways of exposure. *n* is the kinds of trace elements.

### 2.5. Fuzzy Comprehensive Lake Health Assessment Method (FCLHAM)

As one of the most important human habitats, lakes provide a variety of service functions, and their health status is closely related to the survival and development of human beings. How to comprehensively and scientifically evaluate the health status of lakes is becoming an important concern in the field of environmental science and ecology. And it has extremely important application value for the monitoring and management of lakes. Therefore, a fuzzy assessment method needs to be developed to efficiently identify comprehensive lake health states. Based on *TLI*, Health Risk Assessment framework for heavy metals, and fuzzy theory, it is of significance to explore a novel assessment method synthetically considering heavy metals’ health risk, eutrophication risk, and fuzziness of the assessment system. Based on the fuzzy comprehensive evaluation theory [39,40], the comprehensive lake health state was defined as follows:

(12) $$Risk=f\left(Risk{}_{E},Risk{}_{H}\right)\;$$

where *Risk_E_* represents the eutrophication risk of studied lakes, which is assessed by TLI. *Risk_H_* represents the health risk for the heavy metals of studied lakes. And *f* represents the comprehensive lake health state calculation functions.

Fuzzy language recognition theory in fuzzy mathematics was used to identify the risk in this model. The comprehensive lake health state can be calculated as follows:

(13) $$Risk=\tilde{C}\cdot \tilde{R}=\left(C_{1},C_{2}\right)\cdot \left(\begin{array}{c} A_{1}\\ B_{1} \end{array}\begin{array}{c} A_{2}\\ B_{2} \end{array}\begin{array}{c} A_{3}\\ B_{3} \end{array}\begin{array}{c} A_{4}\\ B_{4} \end{array}\right)\;$$

where $\tilde{C}\cdot \tilde{R}$ characterize the *f* in Equation (13). *C* is the weight values of *Risk_E_* and *Risk_H_. C_1_* and *C*_2_ was determined as 0.4 and 0.6 by the Delphi method, which indicated that the risk of lakes depends on the health risk of heavy metals more than the eutrophication risk by expert advices [41,42]. $\tilde{R}$ is membership matrix for levels of *Risk_E_* and *Risk_H_*. *A*_1_, *A*_2_, *A*_3_, *A*_4_, and *A*_5_ represent membership degrees of five levels of *Risk_E_* (Table 1), and *B*_1_, *B*_2_, *B*_3,_ *B*_4_, and *B*_5_ represent membership degrees of five levels of *Risk_H_* (Table 2).

Therefore, the comprehensive lake health state can be represented as a matrix with one row and five columns. The calculated comprehensive lake health state were divided into five levels as follows: (1) level I, low risk; (2) level II, moderate risk; (3) level III, considerable risk; (4) level IV, high risk; (5) level V, very high risk. The membership degree of each assessment factor plays a key role in the fuzzy comprehensive risk assessment, which is the basis of the foundation of comprehensive fuzzy assessment. According to Table 1 and Table 2, the membership function of *Risk_E_* and *Risk_H_* was established, and the membership degree of each level can be calculated by the following formulas [39,40]:

(1) *Risk_E_*

(14) $$u_{1}\left(r\right)=\left\{ \begin{array}{l} 1,r\in \left[0,30\right)\\ \left(50-r\right)/20,r\in \left[30,50\right)\\ 0,r\in \left[50,+\infty \right) \end{array}\right.$$

(15) $$u_{2}\left(r\right)=\left\{ \begin{array}{l} 0,r\in \left[0,30\right)\;or\;\left[60,+\infty \right)\\ \left(r-30\right)/20,r\in \left[30,50\right)\\ \left(60-r\right)/10,r\in \left[50,60\right) \end{array}\right.$$

(16) $$u_{3}\left(r\right)=\left\{ \begin{array}{l} 0,r\in \left[0,50\right)or\left[70,+\infty \right)\\ \left(r-50\right)/10,r\in \left[50,60\right)\\ \left(70-r\right)/10,r\in \left[60,70\right) \end{array}\right.$$

(17) $$u_{4}\left(r\right)=\left\{ \begin{array}{l} 0,r\in \left[0,60\right)\\ \left(r-60\right)/10,r\in \left[60,70\right)\\ 0,r\in \left[70,+\infty \right) \end{array}\right.$$

(18) $$u_{5}\left(r\right)=\left\{ \begin{array}{l} 0,r\in \left[0,70\right)\\ \left(r-70\right)/30,r\in \left[70,100\right)\\ 1,r\in \left[100,+\infty \right) \end{array}\right.$$

(2) *Risk_H_*

(19) $$u_{1}\left(r\right)=\left\{ \begin{array}{l} 1,r\in \left[0,10^{-6}\right)\\ \left(10^{-5}-r\right)/\left(10^{-5}-10^{-6}\right),r\in \left[10^{-6},10^{-5}\right)\\ 0,r\in \left[10^{-5},+\infty \right) \end{array}\right.$$

(20) $$u_{2}\left(r\right)=\left\{ \begin{array}{l} 0,r\in \left[0,10^{-6}\right)\;or\;\left[5\times 10^{-5},+\infty \right)\\ \left(r-10^{-6}\right)/\left(10^{-5}-10^{-6}\right),r\in \left[10^{-6},10^{-5}\right)\\ \left(5\times 10^{-5}-r\right)/\left(4\times 10^{-5}\right),r\in \left[10^{-5},5\times 10^{-5}\right) \end{array}\right.$$

(21) $$u_{3}\left(r\right)=\left\{ \begin{array}{l} 0,r\in \left[0,10^{-5}\right)\;or\;\left[10^{-4},+\infty \right)\\ \left(r-10^{-5}\right)/\left(4\times 10^{-5}\right),r\in \left[10^{-5},5\times 10^{-5}\right)\\ \left(10^{-4}-r\right)/\left(10^{-4}-5\times 10^{-5}\right),r\in \left[5\times 10^{-5},10^{-4}\right) \end{array}\right.$$

(22) $$u_{4}\left(r\right)=\left\{ \begin{array}{l} 0,r\in \left[0,5\times 10^{-5}\right)\\ \left(r-5\times 10^{-5}\right)/\left(10^{-4}-5\times 10^{-5}\right),r\in \left[5\times 10^{-5},10^{-4}\right)\\ 0,r\in \left[10^{-4},+\infty \right) \end{array}\right.$$

(23) $$u_{5}\left(r\right)=\left\{ \begin{array}{l} 0,r\in \left[0,10^{-4}\right)\\ 1,r\in \left[10^{-4},+\infty \right) \end{array}\right.$$

## 3. Results and Discussion

### 3.1. Basic Parameters and Trace Element Concentrations in Surface Water from Studied Lakes

Table 3 provides a statistical summary of the water quality parameters measured in the 21 lakes in Wuhan. The pH of lakes was basically between 9 and 10, and the highest was in L16, the lowest in L1. According to the five levels of standard limited values stipulated in the Chinese Environmental Quality Standard for Surface Water (GB3838-2002), the concentrations of Chl-*a* and EC in lakes were within their target water quality standard. With the exception of L1 and L3, which were slightly below the target, the DO of the other 19 lakes met the target water quality standards. However, the concentrations of TN and TP did not reach the standard. The TP concentrations of 18 of the 21 lakes did not reach the target water quality. There are four lakes (L10; L13; L15, L21) with higher TP concentrations than the Class V water quality standards, far from reaching the target water quality standards of GB3838-2002. The TN concentrations ranged from 2.16 to 5.48 mg·L^−1^, with the highest value in L15 and lowest in L4. Not only did all the studied lakes not meet their target water quality standards, but they also did not reach the limit of the Class V water quality standard of GB3838-2002, indicating that 21 lakes have been heavily polluted with nitrogen and have shown significant eutrophication pollution characteristics. In addition, the SD values of 16 of the 21 lakes did not reach the target water quality standard limit, and the COD_Mn_ concentrations of 12 lakes did not reach the target water quality standard limit, which also showed the serious pollution of lake water.

The detected heavy metal concentrations of 21 lakes in Wuhan are listed in Table 4. Cr was not detected in water samples. The concentration range of As is 1.237–12.148 μg·L^−1^. Except for L20, the concentrations of As in the studied lakes was within the permissible limits of USEPA, WHO, and Chinese Ministry of Health (2007). The concentrations of Cd were all within the permissible limits of China, WHO, and USEPA. The results indicated that the concentration of carcinogenic heavy metals in lakes was not very high, and other related risks of lake health need to be further explored.

### 3.2. Eutrophication State Analysis of the Studied Lakes

The comprehensive trophic level index (TLI) of 21 lakes in Wuhan are calculated and shown in Table S1. L2 and L5 had relatively better water quality with TLI values of 49.14 and 49.44 (corresponding to the medium trophic condition). Unfortunately, most of the studied lakes presented eutrophication to different extents. Particularly, the TLI of lake L15 was higher than the other lakes, which indicated the most severe eutrophication status. The TLI values in the lakes L3, L4, L7, L8, L9, L11, L12, and L19 varied from 49.14 to 72.96, indicating light eutrophication. Furthermore, the other 10 lakes reached medium eutrophication states, decreasing in the order of L13 > L10 > L17 > L6 > L14 > L16 > L1 > L21 > L20 > L18. The main exceeding standard factors of each lake include TN, TP, SD, and COD_Mn_. This result accords with the data listed in Table 3, which indicates that the cause of eutrophication in lakes is the result of the combined effect of reducing substances and nutrients in the water [14,43].

### 3.3. Health Risk Assessment for Heavy Metals in the Studied Lakes

As the results show in Table S2, the values of CR_Cr_ and CR_Cd_ in all lakes were both below 10^−6^, indicating that there was no carcinogenic risk of Cr and Cd. However, there was a certain carcinogenic risk of As because the CR_As_ values of 21 lakes exceeded 10^−6^, decreasing in the order of: medium risk (L20 > L3 > L13 > L6 > L10 > L16) > low risk (L15 > L21 > L8 > L12 > L4 > L14 > L1 > L2 > L11 > L17 > L7 > L9 > L18 > L19 > L5). The maximum and minimum values of CR_As_ were 2.44 × 10^−5^ and 2.48 × 10^−6^, which were 24.4 times and 2.5 times than the lowest risk limit. Moreover, Table S2 indicated that the risk levels of lakes L3, L6, L10, L13, L16, and L20 were Grade III, which indicates that the pollution of carcinogenic heavy metals in these lakes should be given certain attention by relevant local departments. The risk level of the remaining lakes was Grade II, indicating that carcinogenic heavy metal pollution was not very serious, and not a current health risk concern.

### 3.4. Results of the Fuzzy Comprehensive Lake Health Assessment Method (FCLHAM)

To sum up, we can see that some differences surely existed between the results of eutrophication and health risk assessment, which may confuse the decision-maker because these methods unilaterally focus on evaluating the eutrophication level or the health risks of heavy metals of the lakes. For example, the eutrophication level of L15 is very high and belongs to the severely eutrophic category. However, the result of health risk assessment of L15 is low risk. FCLHAM assigns weights to the results of TLI and CR in order to evaluate comprehensive lake health states more comprehensively and accurately. Then, comprehensive lake health state can be calculated. According to *u_i_*(*r*) arithmetic calculation, the assessment matrix is shown in Table 5. The five numbers contained in the assessment matrix represent the degree to which Risk belongs to the level represented by $u_{i}\left(r\right)$. According to the maximum membership principle, the closer the membership of *u_i_*(*r*) to 1, the higher the degree to which Risk belongs to the level represented by *u_i_*(*r*). As is shown in Table 5, average comprehensive lake health state decreased in the sequence of L20 (considerate risk level) > L1~L17, L19, L21 (moderate risk level) > L18 (low risk level). It indicated that L20 needs increased human, material, and financial resource investment and to be given priority regarding its governance. L1~L17, L19, and L21 need more attention, governance, and oversight to maintain their current state. Although L5’s risk level was close to the low risk level, its low risk level (0.508) and moderate risk level (0.492) were too close to each other. Under the principle of maximum risk protection, L5 was determined as moderate risk. If the absolute difference between the memberships of two adjacent risk levels is less than 10%, the final risk level can be determined as the higher level.

Through comparative analysis, we can see: (i) only the heavy eutrophication of L15 also had a high health risk for heavy metals, with a level II risk assessment, indicating that the risk should be noticed. Comparing the results of FCLHAM, L15 belongs to the level II (moderate risk) category, which should attract attention. (ii) All 10 lakes at moderate eutrophication and 8 lakes in mild eutrophication were all rated at level II or level III, while the corresponding results of the FCLHAM levels are also level II or level III, except for L18, whose level was I (low risk). (iii) All three assessment results of L2 and L5, which were in the medium level of nutrition, were level II. That also provided further proof of the reliability of FCLHAM. The first two methods of L5 were level II, and the results of FCLHAM was level I. The examples of L5 and L18 illustrated the more hierarchical and scientific results of FCLHAM than the other two evaluation methods, which makes up for the deficiency of the deterministic assessment.

### 3.5. Classification and Control Countermeasures of Studied Lakes

**Figure 2:**
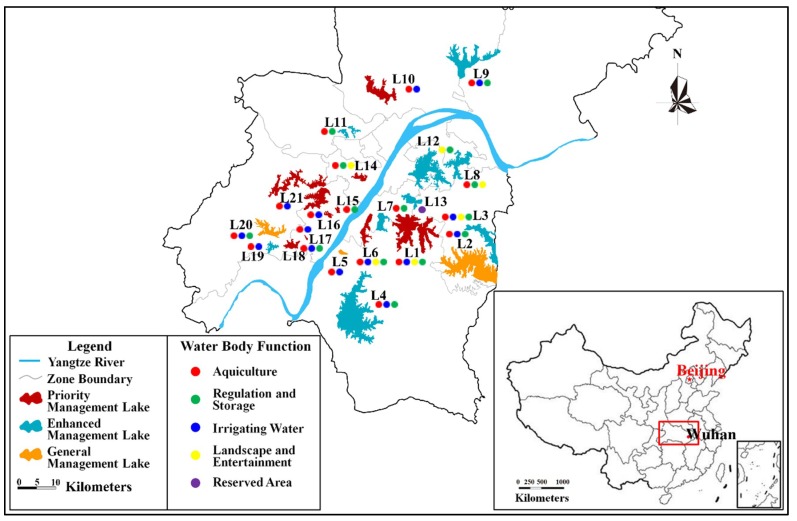
Management classification chart of 21 investigated lakes (ArcGIS software version 9.3 (https://www.arcgis.com/index.html)).

It is understood that lakes in Wuhan are currently subject to cross-sectoral management by functional classification. With the exception of the water sector, other departments, such as fishery, transportation, environmental protection, health, land, forestry, tourism, health, and other related departments, have a certain management function for lakes. However, the pollution treatment of lakes depends on the management and promotion of the environmental protection department, so it is necessary to consider the lakes’ function and nutritional status in the lake classification. Based on the results of FCLHAM, the 21 studied lakes were classified into three categories: general management, enhanced management, and priority management (Figure 2). Figure 2 was made based on the results of FCLHAM with ArcGIS software version 9.3 (https://www.arcgis.com/index.html). The low risk lake (L18) which was rated level I corresponds to common management; enhanced management corresponds to the moderate risk lakes (L1–L17, L19, L21); and priority management corresponds to the considerable risk lake (L20).

According to the characteristics of each kind of lake, we give countermeasures of lake management, specifically: for the common management lakes, its eutrophication level and health risk for heavy metal are both low, so at present, it does not need a great deal of manpower and material resources for remediation, but observation and supervision policies should be implemented so that the lake stays in good condition. The enhanced management lakes, whose risk is in the middle, should be given attention and some treatment measures should be taken. The priority management lakes, whose levels of eutrophication and the health risks for heavy metals are both high, require greater human, material, and financial resource expenditure for remediation to prevent threats to the physical health of the surrounding residents. In addition, the corresponding policies and the necessary source investigation should also be carried out to protect the lake after treatment.

**Figure 3:**
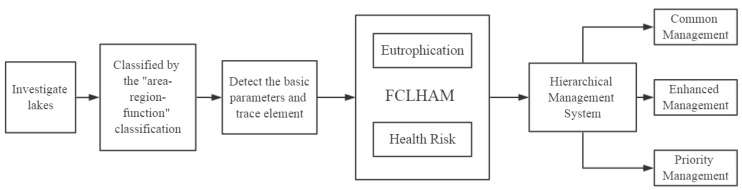
Workflow of the established hierarchical management system.

If the 143 lakes in Wuhan were classified by the “area-region-function” classification, they would be assigned to the same category as the representative lakes of the same type. At this point, we will attribute all of Wuhan’s lakes to the three types of common management lakes, enhanced management lakes, and priority management lakes. Depending on the characteristics of each type, a targeted approach to different types of management for each type of lake is a more efficient way to manage many of Wuhan’s lakes. Based on FCLHAM, a novel hierarchical management system for urban lake health based on lake characteristics classification was obtained (Figure 3). Here, we have tested and verified the rationality, efficiency, and science of this novel hierarchical management system for innovative lake management of Wuhan. Therefore, this management mode can serve as an effective reference for the environmental management of urban lakes both at home and abroad, to manage urban lake health hierarchically and efficiently.

## 4. Conclusions

FCLHAM was established to evaluate lake health states more comprehensively and accurately by integrating quantitative eutrophication and health risk considerations. To test and verify FCLHAM, Wuhan was taken as an example to carry out a practical test. The state of eutrophication and the health risk posed by heavy metals in 21 of the 143 lakes in Wuhan city were investigated and analyzed. Under two different evaluation methods, the results of the same lake were different, and some even deviated greatly, such as L15 and L20, which is very disadvantageous for the managers of urban lake health to administer effective lake management. FCLHAM solves this problem for decision makers and offers the evaluation results of comprehensive consideration of the state of eutrophication and the health risk posed by heavy metals. The evaluation results of FCLHAM are as follows: L20 (considerate risk level) > L1–L17; L19; L21 (moderate risk level) > L18 (low risk level). According to the results, the studied lakes were grouped into three categories (general management lakes, enhanced management lakes, and priority management lakes) and effective protection and management measures were provided with respect to the characteristics of each type of lake. According to the characteristics of lake clustering, all lakes in Wuhan are classified into the above-mentioned three types. According to the characteristics of each type, solutions are put forward for the management each kind of lake. Therefore, FCLHAM offers a novel hierarchical management system for urban lake health based on lake characteristics classification, which can serve as an effective reference for the environmental management of urban lakes both at home and abroad.

## Figures and Tables

**Table 1:** Classification of eutrophication levels.

TLI(Ʃ)	[0, 30)	[30, 50]	(50, 60]	(60, 70]	(70, 100)
Grades	I	II	III	IV	V
Trophic state	Oligotrophic	Medium trophic	Light eutrophication	Medium eutrophication	Severe eutrophication

**Table 2:** Levels and values of assessment standards.

Risk Grades		Range of *CR*	Acceptability
Grade I	Extremely low risk	<10^−6^	Completely accept
Grade II	Low risk	[10^−6^, 10^−5^)	Do not mind about the risk
Grade III	Low-medium risk	[10^−5^, 5 × 10^−5^)	Care about the risk
Grade IV	Medium risk	[5 × 10^−5^, 10^−4^)	Care about the risk and willing to invest
Grade V	High risk	>10^−4^	Pay attention to the risk and take action to solve it

**Table 3:** Basic parameters and target water quality of 21 lakes in Wuhan.

Lake		pH	DO (mg·L^−1^)	Cond (μs·cm^−1^)	Chl-*a* (mg·m^−3^)	TP (mg·L^−1^)	TN (mg·L^−1^)	SD (m)	COD_Mn_ (mg·L^−1^)	Target Quality
L1		8.94	4.41	409.33	4.51	0.19	3.84	0.12	6.94	III
L2		9.07	6.76	147.80	1.10	0.03	2.29	0.25	6.23	II
L3		9.10	4.14	228.50	1.45	0.08	2.50	0.25	7.15	II
L4		9.33	8.24	239.00	1.62	0.04	2.16	0.27	5.86	II
L5		9.46	8.99	217.80	0.44	0.07	3.13	0.23	4.65	/
L6		9.54	11.23	332.50	9.74	0.24	3.96	0.15	5.50	III
L7		9.46	6.50	279.00	0.44	0.18	3.65	0.21	7.65	III
L8		9.40	8.02	322.50	2.40	0.06	2.26	0.20	6.66	III
L9		9.48	10.34	172.20	0.69	0.11	2.42	0.24	6.08	III
L10		9.58	16.98	312.50	8.27	0.25	3.61	0.11	6.62	III
L11		9.78	13.17	336.00	2.12	0.18	3.56	0.24	6.46	IV
L12		9.96	14.53	276.00	5.29	0.12	3.33	0.25	6.31	III
L13		9.90	12.00	506.50	7.39	0.38	4.05	0.19	8.19	IV
L14		9.56	9.10	335.50	9.09	0.15	3.96	0.14	7.27	IV
L15		9.88	14.23	414.00	6.53	0.44	5.48	0.05	8.58	IV
L16		10.02	12.55	293.00	6.36	0.13	3.65	0.10	7.42	IV
L17		9.74	13.09	321.00	14.28	0.15	2.48	0.10	7.73	/
L18		9.64	11.50	326.00	7.72	0.03	2.56	0.09	7.35	IV
L19		9.64	9.35	244.00	1.32	0.01	4.80	0.15	6.58	III
L20		9.50	8.30	246.00	3.02	0.16	3.44	0.14	7.19	III
L21		9.67	11.37	313.50	3.80	0.22	3.12	0.13	6.92	III
Chinese standards ^a^	Class I	6–9	≥7.5	≤2000	≤1.0	≤0.01	≤0.2	≥15.0	≤2.0	/
	Class II		≥6.0		≤4.0	≤0.025	≤0.5	≥4.0	≤4.0	
	Class III		≥5.0		≤10	≤0.05	≤1.0	≥2.5	≤6.0	
	Class IV		≥3.0		≤50	≤0.1	≤1.5	≥1.5	≤10	
	Class V		≥2.0		≤65	≤0.2	≤2.0	≥0.5	≤15	

^a^ The standard values of the Chinese Environmental Quality Standards for Surface Water (GB3838-2002).

**Table 4:** The concentrations of heavy metals in 21 lakes in Wuhan.

Lake	Cr (μg·L^−1^)	As (μg·L^−1^)	Cd (μg·L^−1^)
L1	<0.1 ^b^	3.353	0.033
L2	<0.1	3.165	0.056
L3	<0.1	6.133	0.012
L4	<0.1	3.521	0.035
L5	<0.1	1.237	0.014
L6	<0.1	5.759	0.022
L7	<0.1	2.359	0.017
L8	<0.1	3.951	0.015
L9	<0.1	2.194	<0.1
L10	<0.1	5.534	0.01
L11	<0.1	3.057	0.009
L12	<0.1	3.872	0.004
L13	<0.1	5.812	0.02
L14	<0.1	3.516	0.018
L15	<0.1	4.792	0.021
L16	<0.1	5.095	0.026
L17	<0.1	2.941	0.005
L18	<0.1	2.089	0.021
L19	<0.1	2.062	0.009
L20	<0.1	12.148	0.161
L21	<0.1	4.125	0.005
WHO ^a^	50	10	3
USEPA ^b^	100	10	5
Chinese standards ^c^	50	10	5

^a^ WHO, 2008; ^b^ USEPA, 2009; ^c^ Chinese Ministry of Health, 2007.

**Table 5:** The fuzzy matrix of *Risk_E_* and *Risk_H_*.

Lake	*Risk_E_*	*Risk_H_*	Risk	Membership Level	
L1	(0,0,0.583,0.417,0)	(0.345,0.655,0,0,0)	(0.207,0.393,0.2332,0.1668,0)	II	Moderate risk
L2	(0.043,0.957,0,0,0)	(0.375,0.625,0,0,0)	(0.2422,0.7578,0,0,0)	II	Moderate risk
L3	(0,0.585,0.415,0,0)	(0,0.941,0.059,0,0)	(0,0.7986,0.2014,0,0)	II	Moderate risk
L4	(0,0.932,0.068,0,0)	(0.307,0.693,0,0,0)	(0.1842,0.7886,0.0272,0,0)	II	Moderate risk
L5	(0.028,0.972,0,0,0)	(0.828,0.172,0,0,0)	(0.508,0.492,0,0,0)	I	Low risk
L6	(0,0,0.484,0.516,0)	(0,0.958,0.042,0,0)	(0,0.5748,0.2188,0.2064,0)	II	Moderate risk
L7	(0,0.443,0.557,0,0)	(0.576,0.424,0,0,0)	(0.3456,0.4316,0.2228,0,0)	II	Moderate risk
L8	(0,0.494,0.506,0,0)	(0.222,0.778,0,0,0)	(0.3732,0.6644,0.2024,0,0)	II	Moderate risk
L9	(0,0.764,0.236,0,0)	(0.622,0.378,0,0,0)	(0.3732,0.5324,0.0944,0,0)	II	Moderate risk
L10	(0,0,0.342,0.658,0)	(0,0.971,0.029,0,0)	(0,0.5826,0.1542,0.2632,0)	II	Moderate risk
L11	(0,0.134,0.866,0,0)	(0.425,0.575,0,0,0)	(0.255,0.3986,0.3464,0,0)	II	Moderate risk
L12	(0,0.052,0.948,0,0)	(0.246,0.754,0,0,0)	(0.1476,0.4732,0.3792,0,0)	II	Moderate risk
L13	(0,0,0.308,0.692,0)	(0,0.956,0.044,0,0)	(0,0.5736,0.1496,0.2768,0)	II	Moderate risk
L14	(0,0,0.485,0.515,0)	(0.317,0.683,0,0,0)	(0.1902,0.4098,0.194,0.206,0)	II	Moderate risk
L15	(0,0,0,0,0.0986)	(0.031,0.969,0,0,0)	(0.0186,0.5814,0,0,0.03944)	II	Moderate risk
L16	(0,0,0.51,0.49,0)	(0,0.991,0.009,0,0)	(0,0.5946,0.2094,0.196,0)	II	Moderate risk
L17	(0,0,0.358,0.642,0)	(0.453,0.547,0,0,0)	(0.2718,0.3282,0.1432,0.2568,0)	II	Moderate risk
L18	(0,0,0.829,0.171,0)	(0.634,0.366,0,0,0)	(0.3804,0.2196,0.3316,0.0684,0)	I	Low risk
L19	(0,0.886,0.114,0,0)	(0.646,0.354,0,0,0)	(0.3876,0.5668,0.0456,0,0)	II	Moderate risk
L20	(0,0,0.99,0.01,0)	(0,0.621,0.379,0,0)	(0,0.3726,0.6234,0.004,0)	III	Considerate risk
L21	(0,0,0.698,0.302,0)	(0.189,0.811,0,0,0)	(0.1134,0.4866,0.2792,0.1208,0)	II	Moderate risk

## Supplementary Materials

The following are available online at http://www.mdpi.com/1660-4601/15/12/2617/s1, Table S1: Eutrophication evaluation results of 21 studied lakes, Table S2: Health risk indicators for carcinogenic health for 21 studied lakes, Figure S1: Distribution map of lake in Wuhan city, Figure S2: The workflow of typical lake selection method.
