# Supplementary material for: A Fuzzy Comprehensive Assessment and Hierarchical Management System for Urban Lake Health: A Case Study on the Lakes in Wuhan City, Hubei Province, China
Source: Int J Environ Res Public Health. 2018 Nov 22;15(12):2617. doi: 10.3390/ijerph15122617 (PMC6313550; doi:10.3390/ijerph15122617)
Supplement: Supplementary file 1 [file ijerph-15-02617-s001.pdf]

Supporting information

Table S1. Eutrophication evaluation results of 21 studied lakes

Lake	TLI	Grade	Surpassing factors
L1	64.17	Medium eutrophication	TP、TN、SD、COD_{Mn}
L2	49.14	Medium trophic	TP、TN、SD、COD_{Mn}
L3	54.15	Light eutrophication	TP、TN、SD、COD_{Mn}
L4	50.68	Light eutrophication	TP、TN、SD、COD_{Mn}
L5	49.44	Medium trophic	/
L6	65.16	Medium eutrophication	TP、TN、SD
L7	55.57	Light eutrophication	TP、TN、SD、COD_{Mn}
L8	55.06	Light eutrophication	TP、TN、SD、COD_{Mn}
L9	52.36	Light eutrophication	TP、TN、SD、COD_{Mn}
L10	66.58	Medium eutrophication	TP、TN、SD、COD_{Mn}
L11	58.66	Light eutrophication	TP、TN、SD
L12	59.48	Light eutrophication	TP、TN、SD、COD_{Mn}
L13	66.92	Medium eutrophication	TP、TN、SD
L14	65.15	Medium eutrophication	TP、TN、SD
L15	72.96	Severeeutrophication	TP、TN、SD
L16	64.90	Medium eutrophication	TP、TN、SD
L17	66.42	Medium eutrophication	/
L18	60.10	Medium eutrophication	TP、SD
L19	51.14	Light eutrophication	TN、SD、COD_{Mn}
L20	61.71	Medium eutrophication	TP、TN、SD、COD_{Mn}
L21	63.02	Medium eutrophication	TP、TN、SD、COD_{Mn}

Table S2. Health risk indicators for carcinogenic health for 21 studied lakes.

Lake	CR_i			CR	Grade
	Cr	As	Cd		
L1	<0.1	6.73E-06	1.63E-07	6.89E-06	II
L2		6.35E-06	2.76E-07	6.63E-06	II
L3		1.23E-05	5.92E-08	1.24E-05	III
L4		7.07E-06	1.73E-07	7.24E-06	II
L5		2.48E-06	6.91E-08	2.55E-06	II
L6		1.16E-05	1.09E-07	1.17E-05	III
L7		4.73E-06	8.39E-08	4.82E-06	II
L8		7.93E-06	7.40E-08	8.00E-06	II
L9		4.40E-06	<0.1	4.40E-06	II
L10		1.11E-05	4.93E-08	1.12E-05	III
L11		6.13E-06	4.44E-08	6.18E-06	II
L12		7.77E-06	1.97E-08	7.79E-06	II
L13		1.17E-05	9.87E-08	1.18E-05	III
L14		7.06E-06	8.88E-08	7.14E-06	II

L15	9.62E-06	1.04E-07	9.72E-06	II
L16	1.02E-05	1.28E-07	1.04E-05	III
L17	5.90E-06	2.47E-08	5.93E-06	II
L18	4.19E-06	1.04E-07	4.30E-06	II
L19	4.14E-06	4.44E-08	4.18E-06	II
L20	2.44E-05	7.94E-07	2.52E-05	III
L21	8.28E-06	2.47E-08	8.30E-06	II

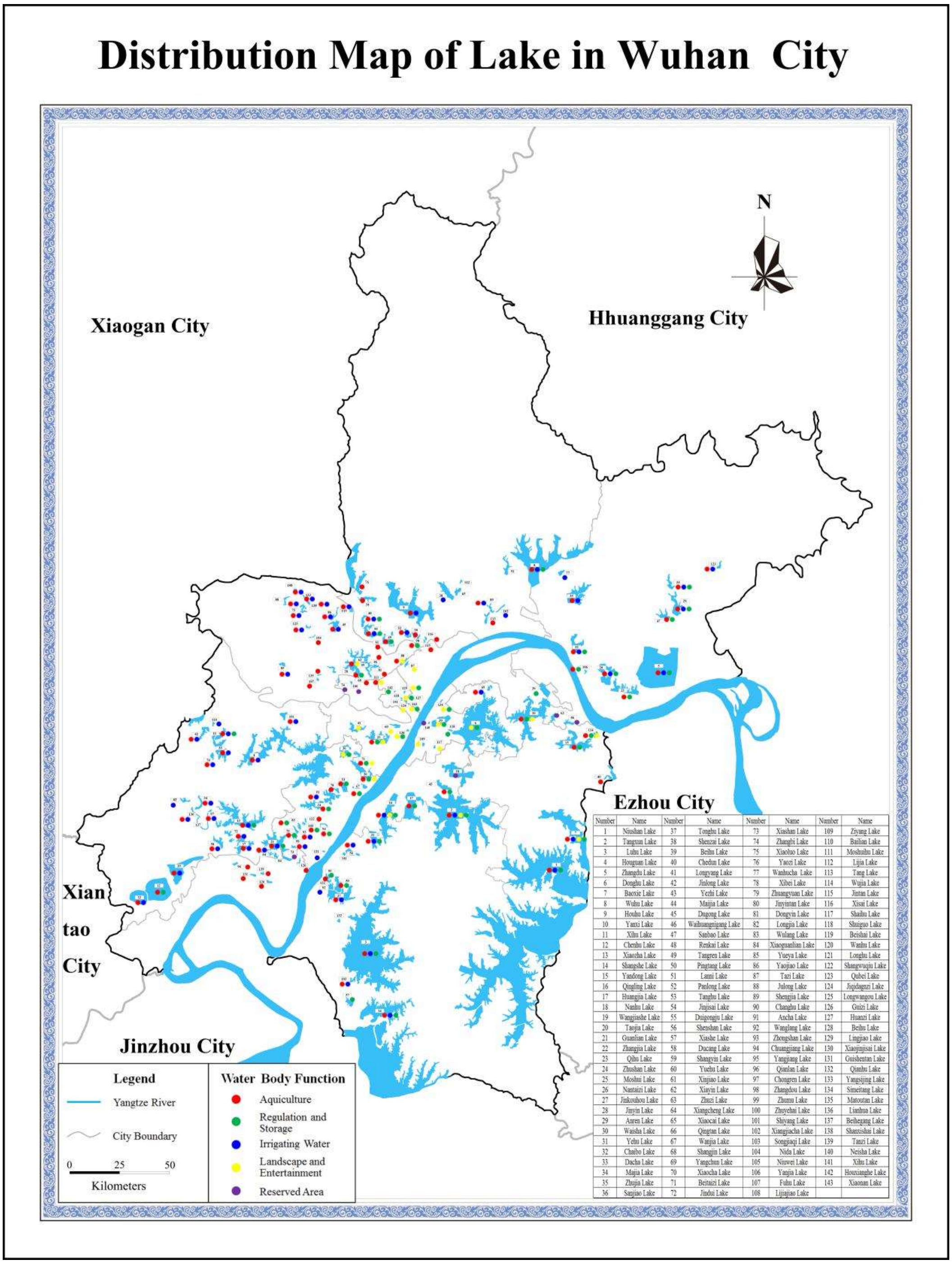

Fig. S1. Distribution map of lake in Wuhan city

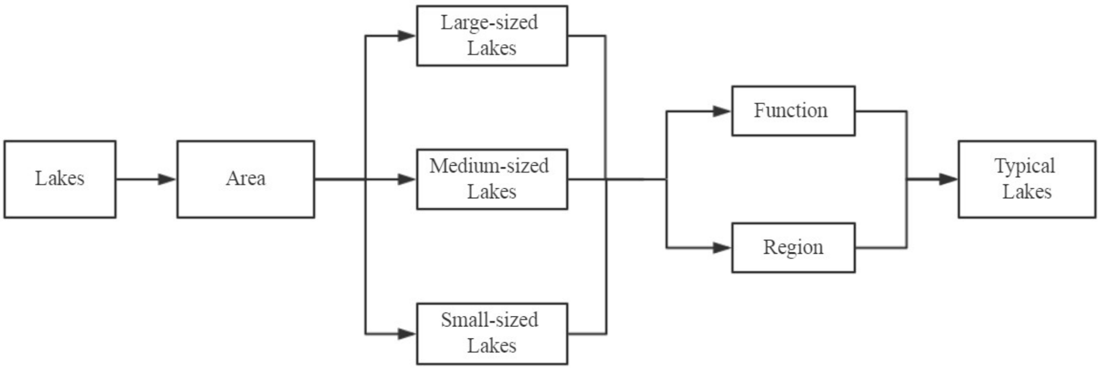

Fig. S2. The workflow of typical lake selection method
